# Antitumour activity of XR5944 *in vitro* and *in vivo* in combination with 5-fluorouracil and irinotecan in colon cancer cell lines

**DOI:** 10.1038/sj.bjc.6602403

**Published:** 2005-02-08

**Authors:** S M Harris, P Mistry, C Freathy, J L Brown, P A Charlton

**Affiliations:** 1 Xenova Ltd, 957 Buckingham Avenue, Slough, Berkshire SL1 4NL, UK; 2 Millennium Pharmaceuticals Inc., 40 Landsdowne Street, Cambridge, MA 02139, USA

**Keywords:** XR5944, 5-fluorouracil, irinotecan, colon cancer, xenografts, combination therapy

## Abstract

XR5944 (MLN944), a novel *bis*-phenazine, has demonstrated potent cytotoxic activity against a variety of murine and human tumour models. In the present study, the antitumour activity of XR5944 was investigated in combination with 5-fluorouracil (5-FU) or irinotecan in human colon carcinoma cell lines and xenografts. *In vitro* cytotoxicity of the combinations following exposure to the drugs sequentially or simultaneously was evaluated by the sulphorhodamine-B assay and interactions were determined using median-effect analysis. Antagonism was observed (CI>1) following exposure of HT29 cells simultaneously to XR5944 and 5-FU or SN38 (active metabolite of irinotecan). In contrast, sequential exposure of either combination in either order demonstrated at least an additive response (CI⩽1). At least an additive response was also observed with these combinations in HCT116 cells regardless of schedule. Antitumour activity in HT29 xenografts in nude mice was enhanced by sequential administration of 5-FU (65 mg kg^−1^) or irinotecan (CPT-11) (35 mg kg^−1^) 48 h before XR5944 (5, 10, or 15 mg kg^−1^) compared to single agent treatment at the same or higher doses. Administration of irinotecan (35 mg kg^−1^) and XR5944 (15 mg kg^−1^) just 30 min apart yielded similar efficacy to sequential administration 48 h apart. All combinations were well tolerated. These data suggest that combinations of XR5944 with irinotecan or 5-FU are of significant interest in the treatment of colon cancer.

XR5944 is a novel *bis*-phenazine that has shown potent cytotoxic activity in a range of human and murine tumour cell lines *in vitro*, as well as significant antitumour activity against human tumour xenografts *in vivo* and *ex vivo* (Cree *et al*, 2001; Gamage *et al*, 2001; Stewart *et al*, 2001). XR5944 has recently entered phase I clinical trials. Although the initial report suggested that XR5944 might act through the joint inhibition of topoisomerase I and II (Stewart *et al*, 2001), other studies have demonstrated that topoisomerases are not the primary cellular targets, and that the compound may work via a novel mechanism of action. For example, XR5944 was evaluated in the NCI human cell line panel (Gamage *et al*, 2001) and COMPARE analysis showed lack of correlation with known topoisomerase poisons and suggested a unique mechanism of action (data not shown). Furthermore, potency of XR5944 was not impaired in human cell lines or in the yeast *Saccharomyces cerevisiae* with reduced levels of topoisomerase enzymes (Gamage *et al*, 2001; Sappal *et al*, 2004). Gene expression profiles of XR5944 and irinotecan-treated human tumour xenografts were distinct, revealing clusters of differentially regulated genes by the two drugs (Sappal *et al*, 2004). Also, XR5944 does not inhibit the catalytic activity of topoisomerase I or II at active concentrations and does not significantly stimulate DNA scission mediated by either topoisomerase I or II unlike camptothecin or etoposide (Sappal *et al*, 2004). Cell cycle effects of XR5944 also indicate mechanisms of action that are distinct from that of known topoisomerase inhibitors. Cells treated with topoisomerase I or II inhibitors such as irinotecan or doxorubicin, respectively, demonstrate characteristic G2-phase cell cycle arrest (Tsao *et al*, 1992; Siu *et al*, 1999; Ueno *et al*, 2002). In contrast, HCT116 colon carcinoma cells treated with XR5944 have shown arrest in both the G1 and G2 phases of the cell cycle (Sappal *et al*, 2003, 2004). Collectively, these data suggest that XR5944 exerts a cytotoxic response principally via mechanisms other than inhibition of topoisomerases I and II.

Colorectal cancer is a major cause of cancer deaths in the developed world. 5-Fluorouracil (5-FU) and irinotecan have to date been the most widely used single agent therapies in the treatment of advanced metastatic colon cancers. Upon cell entry, 5-FU is converted to its active form 5-fluoro-2′-deoxyuridine monophosphate (Allegra and Grem, 1997), whereby a complex is formed with thymidylate synthase (Santi *et al*, 1987), inhibiting its function and impairing DNA synthesis. 5-Fluorouracil is also incorporated into RNA and interferes with RNA processing. It is an S-phase active agent with no activity in G0 or G1 and causes S-phase arrest (Santi *et al*, 1987). Irinotecan (CPT-11) is a water-soluble camptothecin analogue, which inhibits topoisomerase I via conversion to its active metabolite SN38. SN38 inhibits topoisomerase I activity by stabilising the topoisomerase I–DNA cleavable complex, which results in DNA double-strand breaks and ultimately to cell death. Cells in S-phase are significantly more sensitive to camptothecins than cells in G1 or G2 (Li *et al*, 1972), and the compound causes arrest in the G2 phase (Tsao *et al*, 1992).

Combination chemotherapy is now a mainstay in the treatment of advanced and disseminated neoplasia. The rationale for combination therapy is based on the view that resistance to any single agent in a heterogeneous tumour cell population could be overcome by using multiple agents with different mechanisms of action. In addition, rational delivery of combination chemotherapy may induce an additive or synergistic response, for example, by enabling an increase in dose density. It must be noted, however, that overlapping toxicities between combinations may preclude their clinical utility.

The apparently distinct mechanism of action of XR5944 provides a potential opportunity for combination therapy with 5-FU or irinotecan in colorectal cancers. In this study, we show that at least additive cytotoxicity can be achieved by combined treatment of XR5944 with 5-FU or irinotecan (CPT-11), in two human colon carcinoma cell lines (HT29 and HCT116), differing in p53 status and sensitivity to chemotherapeutic drugs. In addition, we demonstrate that this additive cytotoxicity translates to *in vivo* efficacy in the relatively refractory HT29 xenograft, suggesting that promising antitumour activity may be achieved against colorectal cancers by such combination therapy.

## MATERIALS AND METHODS

### Drugs

**Figure 1:**
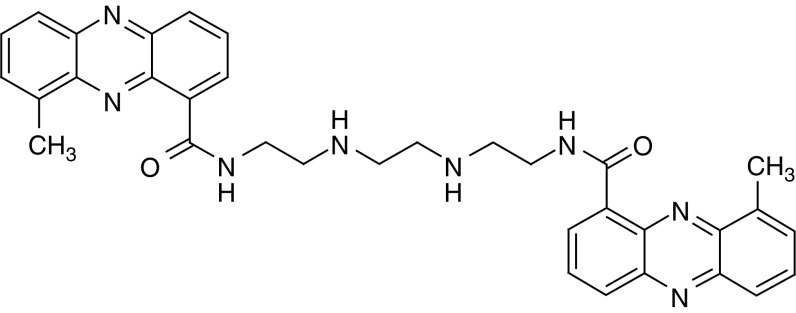
Structure of XR5944.

XR5944 (dimesylate salt) (Figure 1) was synthesised at Auckland Cancer Research Centre and was dissolved in filter-sterilised (0.2 *μ*M) 5% dextrose (w:v). All doses are quoted as free base equivalent. For *in vitro* use, 5-FU was purchased from Sigma (Dorset, UK) and was dissolved in sterile saline. 7-ethyl-10-hydroxycamptothecin (SN38) was dissolved in DMSO prior to use. For *in vivo* use, 5-FU was obtained from Faulding Pharmaceuticals Plc (UK) and CPT-11 (Campto, Irinotecan hydrochloride trihydrate) was obtained from Rhone-Poulenc Rorer (France). Both were diluted in 0.9% sterile saline. All drugs were made up immediately prior to use.

### Cell lines

HT29 and HCT116 human colon carcinoma cell lines were obtained from the ATCC (Rockville, MD, USA). Cells were grown as monolayers under standard conditions in MEM medium supplemented with 1% nonessential amino acids, 1% L-glutamine, 1% sodium pyruvate and 10% FCS in a humidified atmosphere containing 5% CO_2_.

### Cytotoxicity assays

Cells were seeded in 96-well plates at 1 × 10^4^ cells per well for 5-day assays, and 1 × 10^3^ cells per well for 7-day assays 4 h prior to the addition of two-fold serial dilutions of the cytotoxic. These cell densities were chosen so that cells were in exponential growth for the duration of the assay. Analysis of cell growth was assessed by calculation of the IC_50_ values after 5 days. For calculation of the molar ratio for sequential combination assays, IC_50_ values were also calculated following a 48 h incubation of cytotoxic either from days 0–2, or 2–4, with analysis by sulphorhodamine-B (SRB) on day 7. The SRB technique was performed for the determination of the IC_50_ values as described by Skehan *et al* (1990). Briefly, 50 *μ*l ice-cold 50% TCA was added to all wells, and fixed for 1 h at 4°C, washed three times with water, and air-dried. Fixed cells were stained with 50 *μ*l of 0.4% SRB in 1% acetic acid solution for 30 min at RT. After washing three times in 1% acetic acid and air-drying, SRB was solubilised in 100 *μ*l per well 10 mM unbuffered Tris. OD was measured at 510 nm and growth inhibitions were determined relative to untreated cells. For *in vitro* combination assays, cytotoxics were incubated with cells both individually, and together at the ratio of their IC_50_ values as a series of two-fold dilutions from 8 to 0.0625 times IC_50_. Combination assays were performed as a simultaneous schedule (5-day incubation followed by analysis), or sequential schedules (two 48 h incubations followed by analysis on day 7). All assays were carried out in duplicate, and data presented are the mean of at least three independent experiments.

### Median-effect analysis

The combined effect of XR5944 and 5-FU or SN38 treatment was analysed by median-effect analysis according to the method of Chou and Talalay (1984). Combination index (CI) values were expressed at each fraction affected (Fa) using CalcuSyn software (Biosoft) developed by Chou and Chou. CI<1 indicates synergism, CI=1 indicates additivity, and CI>1 indicates antagonism of the interaction. The linear regression coefficient was automatically generated for each assay and was greater than 0.95 in each case.

### Animals

All animal experimentation was performed according to UK Home Office regulations and the UKCCCR guidelines were adhered to throughout the studies. Female CD1 nude mice were purchased from Charles River UK. Animals were maintained under constant temperature and humidity and 12 h light and dark cycle with food and water available *ad libitum*.

### *In vivo* combination studies

HT29 cells were harvested from *in vitro* incubation and were inoculated subcutaneously at 3 × 10^6^ per animal in 100 *μ*l PBS into the right flanks of CD1 athymic mice. When tumours had reached a mean diameter of 5–8 mm (day 0), the animals were randomised into groups of seven and treated by intravenous (i.v.) injection at 10 ml kg^−1^. Body weights and two perpendicular diameters of the tumours were measured at least three times per week. Each tumour volume was calculated according to the following equation: *v*=0.5236[(*l*+*w*)/2]^3^, where *l* and *w* are the largest and smallest perpendicular diameters. Tumour volume and body weights were expressed as mean±s.e.m. relative to tumour volume or body weight values on day 0 (start of treatment). The T/C% ratio (mean relative tumour volume of the treated tumours/mean relative volume of control group × 100) was calculated each time the tumours were measured. The lowest value is expressed as the optimal T/C% for each group. Statistical analysis was performed using two-way analysis of variance (ANOVA) with Bonferroni post-tests.

To evaluate the interaction between XR5944 and 5-FU, 5-FU (65 mg kg^−1^) was administered first on day 0 followed by XR5944 (5 or 10 mg kg^−1^) 48 h later (day 2). This cycle was repeated on days 7 and 14. To evaluate the effects of individual drugs, animals were dosed with 5-FU (65 or 85 mg kg^−1^) on day 0 and vehicle on day 2, or vehicle on day 0 followed by XR5944 (10 or 15 mg kg^−1^) on day 2. Control animals were dosed with vehicle alone. All the cycles were repeated on days 7 and 14.

The interaction between XR5944 and CPT-11 was evaluated using two schedules, where CPT-11 (35 mg kg^−1^) was administered on day 0 followed by XR5944 (15 mg kg^−1^) 30 min (simultaneous schedule) or (10 or 15 mg kg^−1^) 48 h later (sequential schedule). To evaluate the effects of individual drugs, animals were dosed with XR5944 (5, 10, or 15 mg kg^−1^) or CPT-11 (12, 23, or 35 mg kg^−1^) on day 0 and the control animals were dosed with vehicle alone. All the cycles were repeated on days 7 and 14.

## RESULTS

### Cytotoxicity of XR5944, 5-FU and SN38 in HT29 and HCT116 cells

IC_50_ values for HT29 and HCT116 cells incubated for 5 days with XR5944, 5-FU, and SN38 are shown in Table 1. XR5944 was significantly more potent than either 5-FU or SN38 in these cell lines. These values were used to generate the fixed ratios for the simultaneous exposure combination studies. Cytotoxicity assays were also performed using either a 0–48 h or a 48–96 h exposure, followed by analysis on day 7, for the generation of fixed ratios for the sequential exposure combination studies (data not shown).

### Median-effect analysis of XR5944 in combination with 5-FU or SN38

**Figure 2:**
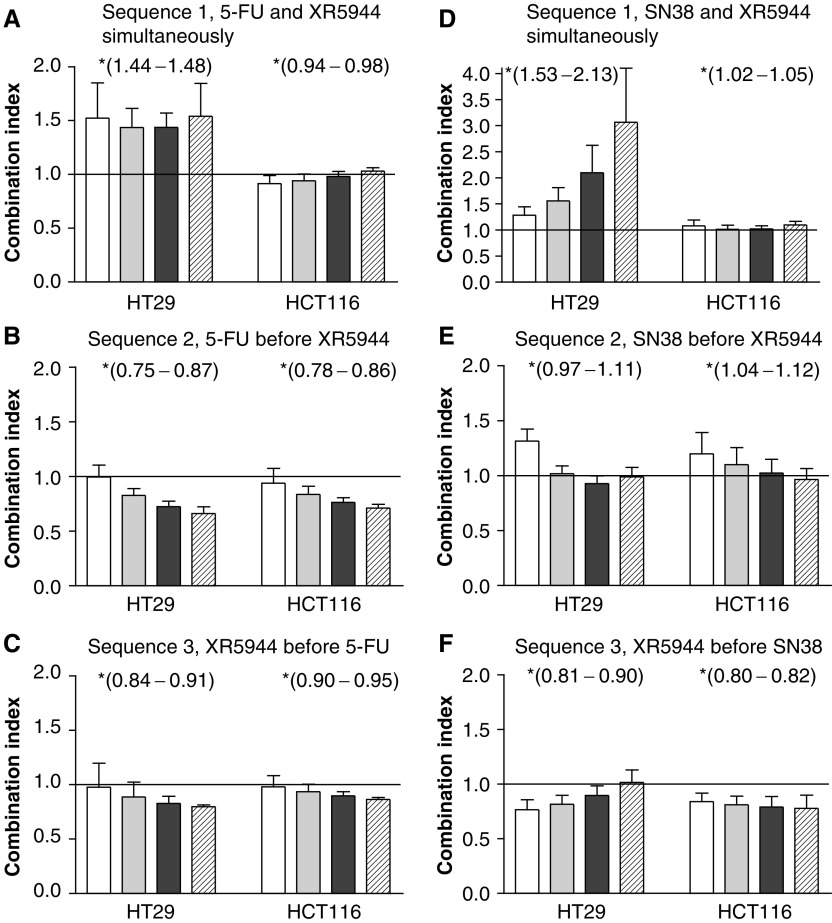
Median effect analysis of the interaction between XR5944 and 5-FU or SN38 in the HT29 and HCT116 cell lines. Sequence 1, simultaneous incubation for 5 days; sequence 2, 5-FU or SN38 exposure for 48 h, and then XR5944 exposure for 48 h; sequence 3, XR5944 exposure for 48 h, and then 5-FU or SN38 exposure for 48 h. □ Fa 0.25, 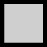 Fa 0.5, 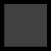 Fa 0.75, and 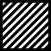 Fa 0.9. CI is plotted as a function of the fraction of cells affected by the cytotoxic effect (Fa). CI>1.1 indicates antagonism, CI=0.9–1.1 indicates additivity and CI<0.9 indicates synergism. Values are the means of three independent experiments±s.e.m. ^*^95% confidence intervals of the mean were calculated for the range Fa 0.25–0.9 (data points at 0.05 intervals) and are shown in brackets.

Figure 2 shows the median effect analysis of XR5944 in combination with 5-FU or SN38 in HT29 and HCT116 cells assuming mutual exclusivity of the interactions. Simultaneous exposure of HT29 cells to XR5944 and 5-FU yielded CI values greater than 1 over the entire range of cytotoxicity (Figure 2A) indicating a less than additive effect (95% confidence interval of the mean 1.44–1.48 at Fa 0.25–0.9). The CI was 1.4 at 50% growth inhibition, indicating that the amount of the two drugs required to kill 50% of cells was 1.4 times as much as would be required if they demonstrated additive behaviour. In contrast, when these cells were sequentially exposed to the two drugs in either order (Figure 2B and C), an additive to synergistic effect was observed and this was particularly notable when 5-FU was added first (CI=0.66±0.06 at Fa 0.9, and 95% confidence interval of 0.75–0.87 at Fa 0.25–0.9). Likewise in the HCT116 cell line, although simultaneous exposure showed additive effects (Figure 2A), sequential exposure of 5-FU before XR5944 improved the efficacy of the two drugs in combination (Figure 2B).

Simultaneous exposure of HT29 cells to XR5944 and SN38 also demonstrated antagonistic effects over the entire range of cytotoxic activity (Figure 2D) and was particularly marked at high fractional effects (CI=3.1 at 90% growth inhibition, 95% confidence interval of 1.53–2.13 at Fa 0.25–0.9), whereas following sequential exposure to the two drugs in either order, CI values at mid- to high fractional effects were close to 1, indicating additivity (Figure 2E and F). In the HCT116 cell line, however, this combination showed additivity at mid- to high fractional effects using both the simultaneous and the sequential schedules (CI=0.8–1.2).

### Antitumour activity of XR5944 and 5-FU alone and in combination against the HT29 xenograft in nude mice

**Figure 3:**
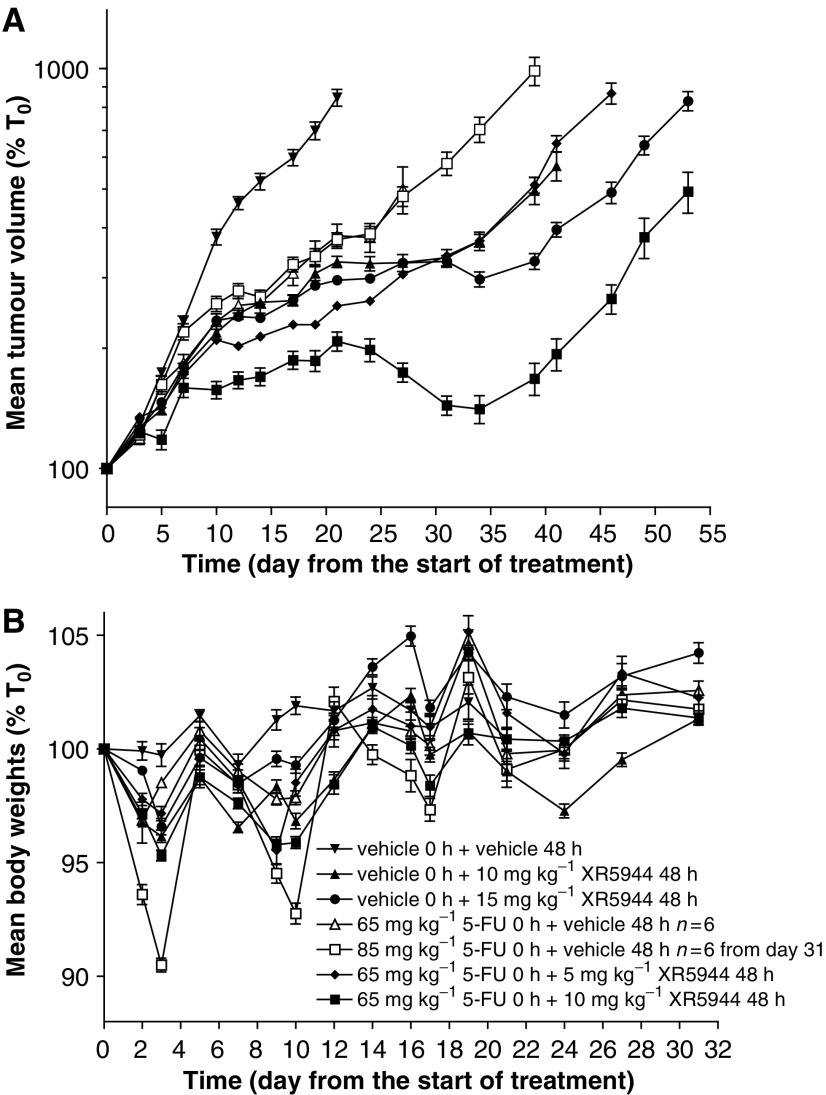
Antitumour activity of 5-FU and XR5944 alone and in combination against the HT29 human colon carcinoma xenograft. (**A**) Tumour volume plotted as a percentage of that on day 0. (**B**) Animal body weight plotted as a percentage of that on day 0. All solutions were administered i.v. at 10 ml kg^−1^ using a q7dx3 dosing schedule and starting on day 0. 5-Fluorouracil was administered on days 0, 7, and 14, and XR5944 on days 2, 9, and 16. Data are expressed as means±s.e.m. *n*=7 except where indicated (where group size was changed partway through the study, this was due to tumour reaching maximum permitted diameter).

To further investigate the therapeutic potential of combining XR5944 with 5-FU, we studied activity of this combination against the relatively refractory HT29 human colon carcinoma xenograft in nude mice using the most favourable *in vitro* schedule (5-FU followed by XR5944 at 48 h). XR5944 alone at 10 or 15 mg kg^−1^ showed significant (*P*<0.001) antitumour activity against the HT29 tumours, at doses well below the MTD for this compound (22.5 mg kg^−1^). In addition, a dose-dependent response was observed particularly from day 31 onwards (Figure 3A), and both doses were well tolerated as indicated by lack of significant body weight loss compared with control animals (Figure 3B).

Animals treated with 5-FU at 65 or 85 mg kg^−1^ showed significant tumour growth inhibition compared to those given vehicle (Figure 3A). However, no dose response was observed (optimal T/C% ratios 49.7 and 51.4, for 65 and 85 mg kg^−1^, respectively) and 85 mg kg^−1^ dose caused significant (10%) body weight loss and was considered to be near MTD. Moreover, the activity of XR5944 at 10 and 15 mg kg^−1^ was significantly (*P*<0.001) greater than that of 5-FU.

Sequential treatment with 65 mg kg^−1^ 5-FU followed by 5 or 10 mg kg^−1^ XR5944 48 h later showed a dose-dependent response (Figure 3A) (optimal T/C% ratios 38.2 and 31.1, respectively). Both combination doses were well tolerated with maximum body weight loss of 4.5 and 4.7%, respectively (Figure 3B). From day 17, significantly (*P*<0.001) better efficacy was observed with 65 mg kg^−1^ 5-FU plus 5 mg kg^−1^ XR5944 than with 5-FU alone at the highest dose of 85 mg kg^−1^ (Figure 3A). Likewise, this combination showed significantly (*P*<0.01) better antitumour activity than the highest dose of XR5944 alone (15 mg kg^−1^) from day 10 to 31, after which time the group dosed with XR5944 alone showed better activity (Figure 3A).

Treatment with 65 mg kg^−1^ 5-FU followed by 10 mg kg^−1^ XR5944 48 h later led to significantly better efficacy than with either 5-FU or XR5944 alone or the sequential combination of 5-FU and 5 mg kg^−1^ XR5944 (*P*<0.001) from day 10. Regression was observed following the third dose cycle (days 14 5-FU and 16 XR5944) (Figure 3A) when mean tumour volume decreased from 208% of start volume on day 21 to 141% on day 34.

### Antitumour activity of XR5944 and CPT-11 alone and in combination against the HT29 xenograft in nude mice

**Figure 4:**
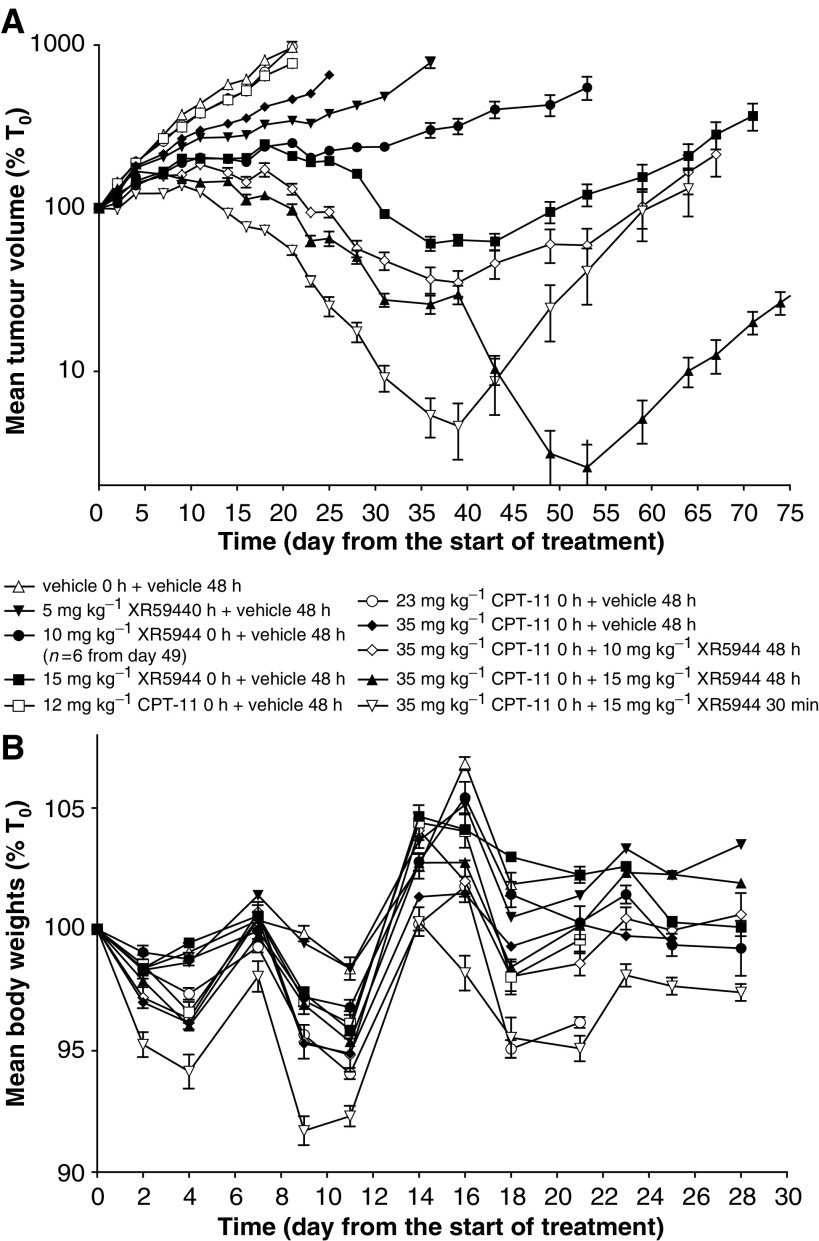
Antitumour activity of CPT-11 and XR5944 alone and in combination against the HT29 human colon carcinoma xenograft. (**A**) Tumour volume plotted as a percentage of that on day 0. (**B**) Animal body weight plotted as a percentage of that on day 0. All solutions were administered i.v. at 10 ml kg^−1^ using a q7dx3 dosing schedule and starting on day 0. Data are expressed as means±s.e.m. *n*=7 except where indicated (one animal was removed from the study on day 49 as the tumour had reached maximum permitted diameter).

Antitumour activity *in vivo* following combination therapy with CPT-11 and XR5944 was evaluated in the HT29 xenograft following simultaneous and sequential administration (30 min and 48 h apart between drug administration, respectively). XR5944 alone at 5, 10, or 15 mg kg^−1^ showed significant (*P*<0.001) and dose-dependent (optimal T/C% ratios 35.5, 25.9, and 21.6, respectively) antitumour activity against the HT29 tumours (Figure 4A). These doses of XR5944 were well tolerated and no significant body weight loss was observed compared to control animals (Figure 4B). XR5944 (15 mg kg^−1^) led to tumour regression, but all tumours regrew. The response of XR5944 alone in this study was more favourable than in the 5-FU study. This was most likely due to the smaller tumour size at the start of XR5944 treatment in this study (dosed starting on day 0 compared to XR5944 in the 5-FU study, which was dosed starting on day 2).

Animals treated with CPT-11 at 12 or 23 mg kg^−1^ did not show significant growth inhibition (*P*>0.05) compared to vehicle treated tumours until day 14 (optimal T/C% ratios 80.0 and 82.1). Treatment with 35 mg kg^−1^ (105 mg m^−2^) CPT-11 caused significant (*P*<0.01) tumour growth delay by day 7 and the T/C% ratio was 48.2. However, the antitumour activity of XR5944 was significantly greater than that of CPT-11. All doses of CPT-11 were well tolerated in this study (Figure 4B) and were lower than the reported MTD of CPT-11 (100 mg kg^−1^ i.v. in mice given as a one off dose or on a weekly schedule; Okuno *et al*, 2000; Cao *et al*, 2004).

Both sequential treatment schedules (35 mg kg^−1^ CPT-11 followed by 10 or 15 mg kg^−1^ XR5944 48 h later) showed significantly (*P*<0.001) greater efficacy than single agent treatment at the same doses. Treatment with 35 mg kg^−1^ CPT-11 followed by either 10 or 15 mg kg^−1^ XR5944 48 h later led to complete tumour regression in four or six animals, respectively, although in both groups half the tumours regrew by day 60.

Treatment with 15 mg kg^−1^ XR5944 30 min after 35 mg kg^−1^ CPT-11 (simultaneous schedule) showed a faster rate of tumour regression than when the drugs were administered 48 h apart (Figure 4A) (T/C% ratios 5.7 and 10.2, respectively, on day 21). This is probably due to the difference in tumour volume when XR5944 was dosed day 0 for simultaneous and day 2 for sequential schedules. However, the overall outcome was similar between the simultaneously and sequentially dosed groups and they were not significantly different (*P*>0.05). Using the simultaneous schedule, six animals showed complete tumour regression, but two of these regrew. All combinations were well tolerated, but simultaneous dosing led to more body weight loss (maximum 8.3% (35 mg kg^−1^ CPT-11+15 mg kg^−1^ XR5944)) than sequential dosing (maximum 5.2% (35 mg kg^−1^ CPT-11+10 mg kg^−1^ XR5944)) or single agent dosing (maximum 5.9% (23 mg kg^−1^ CPT-11)) (Figure 4B).

## DISCUSSION

In this study, XR5944 was found to be more potent *in vitro* than either 5-FU or SN38 in both HT29 and HCT116 cells. XR5944 also exhibited superior *in vivo* potency and efficacy in the HT29 xenografts in nude mice compared with 5-FU or CPT-11 alone. These data confirm previous studies, which show that XR5944 has single agent potency against human cell lines, with an *in vitro* IC_50_ in the range 0.04–0.4 nM (Stewart *et al*, 2001). Furthermore, XR5944 is similarly potent against serially transplanted HT29 xenografts and additionally elicits tumour regression against this model using a daily administration schedule at well-tolerated doses (data not shown). This remarkable efficacy together with a potential novel mechanism of action and preclinical safety profile has provided a great deal of support for XR5944 to enter phase I clinical trials in solid tumours.

Combination chemotherapy is a classical approach to improving chemotherapeutic efficacy in cancer patients compared to single agent treatment. The aim of this study was to investigate the potential for use of XR5944, an agent with a possible novel mechanism of action in combination with the commonly used chemotherapeutic agents 5-FU and CPT-11 for the treatment of colon cancer. In *in vitro* combination studies, simultaneous addition of XR5944 and 5-FU to HT29 cells in monolayer culture showed antagonistic activity. However, sequential addition of the two compounds was additive when cells were incubated with one drug for 48 h before exposure to the other. Similarly, antagonism was observed with simultaneous exposure of HT29 cells to XR5944 and SN38 but not with sequential exposure. Conversely, in HCT116 cells, antagonism was not seen with simultaneous exposure of cells to XR5944 and either 5-FU or SN38. These differences in response between HT29 and HCT116 following simultaneous exposure to the drug combinations may be attributable to the p53 status of the cells. The HT29 cells have mutant p53, while HCT116 cells have wild-type p53 (Jia *et al*, 1997; Violette *et al*, 2002) and although it has previously been suggested that the mechanisms of XR5944-mediated cell cycle arrest may be independent of p53 status (Sappal *et al*, 2003), the cell cycle response to both 5-FU and CPT-11 treatment in colon cancer cells has been reported to be influenced by p53 status (Magrini *et al*, 2002; Violette *et al*, 2002). Both 5-FU and irinotecan (CPT-11) are prodrugs, requiring conversion for their action. In addition, significant differences in metabolism and clearance of CPT-11 and its active species, SN38, have been reported to occur between the HT29 and HCT116 cell lines (Cummings *et al*, 2002). Therefore, another possible cause for the observed differences in the response between these cell lines may be differences in metabolism and the extent of drug–drug interaction and hence variation in exposure to active species. Similarly, differences in cellular pharmacology following simultaneous and sequential exposure to XR5944 and SN38 or 5-FU may also be responsible for the observed schedule-dependent response to these agents in the HT29 cells. Indeed differences in cellular pharmacology of 5-FU and CPT-11 have been reported to be responsible for the strong synergism following sequential exposure and only additivity or antagonism after simultaneous exposure to the two drugs in the HT29 cells (Guichard *et al*, 1998). Cell cycle-mediated drug resistance (Shah and Schwartz, 2001) may also explain the antagonistic response observed when HT29 cells were simultaneously exposed to XR5944 and 5-FU or SN38. Both 5-FU and topoisomerase I inhibitors such as irinotecan are highly S phase specific with little or no activity in the G1 phase (Santi *et al*, 1987; Tsao *et al*, 1992; Voigt *et al*, 1998). The predominantly G1 cell cycle arrest caused by XR5944 (Sappal *et al*, 2003) may therefore inhibit the action of these two agents. In sequential exposure assays, the first drug was washed out before the addition of the second drug, perhaps releasing the cells back into the cycle, although preliminary data suggest that the XR5944-induced block is irreversible (Sappal *et al*, 2003). Such cell cycle-mediated drug resistance has also been observed with other drug combinations. For example, simultaneous exposure of HT29 cells to 5-FU and irinotecan demonstrated antagonism, caused by arrest in G2 phase by irinotecan and concomitant resistance to S-phase specific 5-FU (Guichard *et al*, 1997; Mans *et al*, 1999). However, this antagonism could be overcome by sequential exposure to 5-FU followed by irinotecan after a 6 h delay (Guichard *et al*, 1998). Similarly, exposure of L1210 cells to cisplatin followed by the M-phase-specific drug paclitaxel demonstrates antagonism (Rowinsky *et al*, 1993) apparently caused by the arrest of cells in G2 by cisplatin (Sorenson and Eastman, 1988).

*In vivo*, XR5944 alone or in combination with 5-FU or CPT-11 showed significant antitumour activity against HT29 xenografts in nude mice. The increase in activity was indicative of at least additive activity and is in agreement with the *in vitro* studies. Interestingly, the simultaneous administration schedule, where XR5944 was administered 30 min after CPT-11, also showed comparable additive or synergistic activity, whereas *in vitro* simultaneous exposure to these drugs resulted in antagonistic activity against the HT29 cells. The reason for this difference in response is not clear. These results suggest that *in vitro* studies may not predict accurately *in vivo* response. The simultaneous administration schedule caused greater body weight loss than the sequential schedule, suggesting that the therapeutic index is greater following sequential administration and may be generally more tolerable. Additive toxicities are commonly problematic when combining cytotoxics *in vivo*. However, sequential administration of 35 mg kg^−1^ CPT-11 or 65 mg kg^−1^ 5-FU followed by XR5944 showed improved efficacy over single agent treatment without a substantial increase in toxicity as determined by changes in body weights and the absence of diarrhoea. The major toxicity of XR5944 in mice is haematological, including a reduction in red and white blood cells, and in dogs the major toxicity was GI intolerance (data not shown). In man, 5-FU and CPT-11 are known to cause myelosuppression and diarrhoea, depending on schedule (Sobrero *et al*, 1997; Mathijssen *et al*, 2002). Thus, it is possible that combination of XR5944 with either of these agents may lead to increased myelosuppression and/or GI toxicity in man. The fact that XR5944 is equally active when administered at 5 mg kg^−1^, qdx5 schedule as on an intermittent schedule (q7dx3 at 15 mg kg^−1^) in SCLC (Stewart *et al*, 2001) and in colon xenografts (data not shown) suggests that it may be possible to use these drugs in combination at various schedules to overcome such side effects.

Taken together, these data show that XR5944 is a potent cytotoxic agent that can induce at least additive activity in combination with 5-FU or CPT-11 *in vitro* against both p53 mutant and wild-type colon carcinoma cell lines. Importantly, the potency and beneficial effects of XR5944 in combination with 5-FU and CPT-11 were also translated *in vivo*, against the HT29 xenografts. These data suggest that clinical investigation of sequential combinations of these agents is warranted and could be of benefit to patients with refractory colorectal carcinoma.

## Figures and Tables

**Table 1:** Growth inhibition of HT29 or HCT116 cell lines exposed to XR5944, 5-FU, or SN38

	**Cell line (IC_50_, nM)**	
	**HT29**	**HCT116**
XR5944	0.6±0.2	1.0±0.4
5-FU	1616±1100	5635±930
SN38	4.2±0.7	8.4±3.3

5-FU=5-fluorouracil.

Cells were exposed for 5 days and the concentration that inhibits 50% of growth compared to controls was determined. Values are the mean of at least three independent experiments±s.d.
